# The influence of partial public reimbursement on vaccination uptake in the older population: a cross-sectional study

**DOI:** 10.1186/s12889-015-1356-7

**Published:** 2015-02-05

**Authors:** Sheena M Mc Hugh, John Browne, Ciaran O’Neill, Patricia M Kearney

**Affiliations:** Department of Epidemiology & Public Health, Western Gateway Complex, University College Cork, Western Rd, Cork, Ireland; School of Business and Economics, National University of Ireland, Galway, Ireland

**Keywords:** Influenza vaccine, Coverage, Socioeconomic status, Elderly

## Abstract

**Background:**

Flu vaccination is recommended annually for high risk groups. However, in Ireland, free access to vaccination is not universal for those in high risk groups; the vaccine and consultation are only free for those with a medical card, a means tested scheme. Few private health insurance policies cover the cost of attendance for vaccination in general practice. The aim was to examine the influence of this reimbursement policy on vaccination coverage among older adults.

**Methods:**

Cross-sectional wave 1 data from The Irish Longitudinal Study on Ageing (TILDA) were analysed (2009–2011). TILDA is a nationally representative prospective cohort study of adults aged ≥50, sampled using multistage stratified clustered sampling. Self-reported entitlement to healthcare was categorised as 1) medical card only 2) private health insurance only, 3) both and 4) neither. The outcome was responses to ‘have you ever had a flu shot’. Multivariate logistic regression was used, adjusting for age and need.

**Results:**

68.6% of those defined as clinically high-risk received the flu vaccination in the past (95% CI = 67-71%). Those with a medical card were almost twice as likely to have been vaccinated, controlling for age and chronic illness (OR = 1.9, 95% CI = 1.5-2.5, p = <0.001).

**Conclusions:**

Having a medical card increased the likelihood of being vaccinated, independent of age and need. The mismatch between vaccination guidelines and reimbursement policy is creating unequal access to recommended services among high risk groups.

**Electronic supplementary material:**

The online version of this article (doi:10.1186/s12889-015-1356-7) contains supplementary material, which is available to authorized users.

## Background

Seasonal influenza (flu) vaccination is a cornerstone of preventive health care. Older people and those with certain chronic conditions are considered a ‘high risk’ group as they are at increased risk of severe illness, hospitalisation or death if infected with influenza [1]. While there is some debate about the efficacy of the influenza vaccination, it continues to be a key strategy for reducing flu-related morbidity and mortality [2,3].

Achieving complete flu vaccination coverage in high risk groups remains a challenge for many countries [4]. A UK study found that only 61% of those in high risk clinical groups had received the vaccine in 2009/10 [5]. There is substantial variation in uptake rates between countries [6,7]. The Survey on Health Ageing and Retirement in Europe (SHARE) found that vaccination rates among those aged 60 years and over ranged from 11% in Poland to 62% in the Netherlands [7]. The age-standardised average uptake rate was 41%. Results from the first wave of The Irish Longitudinal Study on Ageing (TILDA) indicate that 50% of those aged 50 years and over have received the flu vaccination in the past [8]. The Council of the European Union has set a target of 75% vaccination coverage for at-risk groups by 2015 [9].

As in most other developed countries [10,11], in Ireland annual flu vaccination is recommended for older people (≥65 years), and those with underlying health conditions including chronic respiratory disease, chronic heart disease, diabetes and chronic liver disease. In addition, groups such as carers and those considered morbidly obese (≥40 kg/m^2^) are recommended to receive the vaccine in Ireland [12]. The vaccine is administered in a primary care setting by a General Practitioner (GP), by a practice nurse or, since 2011, in community pharmacies.

Age and clinical need are well-established in the literature as significant predictors of vaccination coverage [6,7,13-15]. Less attention has been paid to the role of socio-economic factors, but there is evidence suggesting that factors such as income can have a significant impact on flu vaccination rates [4], and that the direction of this effect varies among countries [6]. Countries differ in their approach to the financing of and reimbursement for flu vaccination [11].

In Ireland, reimbursement for flu vaccination is limited, covering some but not all of the high risk groups recommended for vaccination. The vaccine and consultation are free for those (within recommended groups) who hold a medical card. Having a medical card entitles the holder to free access to primary and community care services and exemption from co-payments in public hospitals. Medical card eligibility is primarily based on income; to qualify an individual’s weekly income must be below a certain threshold. Approximately 37% of the population have a medical card [16], rising to 90% of those aged 70 years and older [8]. Persons within recommended groups who do not have a medical card must pay a consultation fee for the administration of the vaccine although the vaccine itself is free. The consultation fee ranges from €35 to €70 euro, with an average cost of €51 euro [17]. Some practices offer reduced rates for services such as flu vaccination.

Approximately 53% of those aged 50 years and over in Ireland have private health insurance [8], however few insurance policies cover the cost of GP attendance and a limited number offer reduced fees for vaccinations at private clinics. In Ireland, private health insurance is voluntary and duplicative, that is, it covers services already provided by the public health system but also provides access to other providers (e.g. private hospitals) or to different levels of service (e.g. faster access) [18]. Data from Ireland provide a unique opportunity to investigate the impact of reimbursement policy on vaccination coverage. The aim of this study was to examine the influence of partial reimbursement on flu vaccination uptake among older adults, controlling for the impact of age and clinical need.

## Methods

### Study design and population

Our study is based on 8175 adults aged 50 years and over from wave 1 of The Irish Longitudinal Study on Ageing (TILDA). TILDA is a prospective cohort study conducted with a nationally representative sample of non-institutionalized adults in the Republic of Ireland. Multistage stratified clustered sampling was conducted using the Geodirectory, a list of all the residential addresses in the Republic of Ireland, as the sampling frame. A response rate of 62% was achieved based on the proportion of eligible households from which an interview was successfully obtained (6,279 households). The design and methodology are described in detail elsewhere [19,20]. Ethical approval was obtained from Trinity College Dublin Research Ethics Committee and written informed consent was obtained from participants.

Data were collected between October 2009 and February 2011 using three methods: computer-assisted personal interviewing (CAPI) including questions on socio-demographics, physical and mental health, employment/retirement, and health service use; a Self-Completed Questionnaire (SCQ) containing questions on quality of life, alcohol, anxiety, social connectedness and perceptions of ageing; and a detailed health assessment.

### Measures

The outcome was whether participants had ever received the flu vaccination in the past. In wave 1 of TILDA, the survey question was ‘have you ever received a flu shot’. The independent variable of interest was entitlement to health care categorised as: those with a medical card only, those with private health insurance only, those with both a medical card and private health insurance (defined as dual cover) and those with neither (defined as ‘no additional cover’).

### Statistical analysis

Vaccination coverage was calculated based on the percentage of individuals that reported *ever* having received a flu vaccination. We examined coverage among those in the ‘total high risk group’ recommended for vaccination according to guidelines from the Royal College of Physicians [12]: those aged ≥65 years, those with a long-term condition requiring follow-up (chronic lung disease, asthma, chronic heart disease, diabetes, liver disease, Parkinson’s disease, stroke/TIA), those classified as morbidly obese (≥40 kg/m^2^) and those in receipt of state-provided carers’ allowance. We compared this to coverage among those in the ‘clinical risk group’ only, that is those with a long-term condition requiring follow-up (chronic lung disease, asthma, chronic heart disease, diabetes, liver disease, Parkinson’s disease, stroke/TIA).

Multivariate logistic regression was used to assess the independent influence of entitlement to health services on vaccination uptake adjusting for age, gender, income, education, employment, self-rated health and clinical need. Covariates were selected a priori based on Andersen’s model of health service utilisation which categorises determinants as predisposing (age, sex), enabling (education, income, employment) and need factors (clinical need and self-rated health) [21]. These factors have also been linked to entitlement to health services as those with a medical card are more likely to be older, less likely to be in good health and less likely to be working [8]. Clinical need was grouped into 1) chronic illnesses targeted in the national immunisation guidelines listed above [12], 2) other chronic conditions requiring regular follow-up (arthritis, osteoporosis, cancer, psychological disorders, alcohol abuse, memory impairment, stomach ulcers and varicose ulcers), 3) cardiovascular risk factors (hypertension and high cholesterol), and (4) no chronic illness. We also included other risk groups recommended in the national guidelines for whom data were available (morbid obesity and receipt of carers’ allowance) [12]. Multicollinearity between covariates was examined by calculating the mean and individual covariate variance inflation factors (VIF). None of the individual covariate VIF were greater than 1.5 and the mean variance inflation factors for all covariates included in the models was 1.1. Interaction terms and stratified models were used to investigate interaction between predictors. Adjusted Population Attributable Fraction (PAF) estimates were used to calculate the proportion of vaccination coverage attributable to each level of entitlement to healthcare. The results were weighted to reflect the complex sampling design and analyses were carried out using Stata 12.

## Results

The mean age of the sample was 63.6 years (sd ± 9.3) and 52.1% were female. Overall, 49.9% of the population aged 50 years and over had received the vaccination in the past (95% CI: 49-51%) (n = 4100). In terms of coverage for health services within the sample, 36.4% had a medical card only (95% CI: 35-38%, n = 2621) and 16.1% had both a medical card and private health insurance (dual coverage) (95% CI: 15-17%, n = 1418).

**Figure 1:**
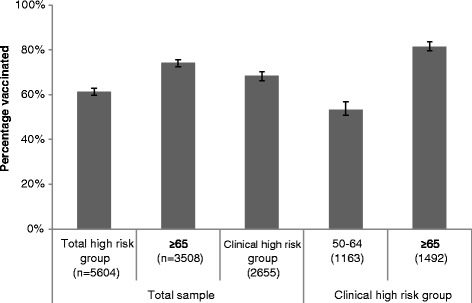
**Vaccination rates across eligibility groups illustrating percentage uptake and 95% confidence intervals around estimates.**

Based on national guidelines, 68.6% of the population were eligible for vaccination at the time of interview (95% CI: 67-70%) (n = 5607) [12]. As Figure 1 illustrates only 61.4% of that group were vaccinated (95% CI: 60-63%) (n = 3441). Among the clinical risk group (those with chronic conditions requiring continued follow-up and excluding eligibility based on age), 68.6% were vaccinated (95% CI: 67-71%). Uptake in the clinical risk group was much higher among those aged ≥65 compared to those aged 50 to 64 years. Of those who did not meet any of the ‘high-risk’ criteria, 17.2% (95% CI: 15-20%) had received the vaccination in the past (n = 184).

Uptake rates within each of the at-risk groups varied according to entitlement status, with higher vaccination rates among medical card holders and those with dual cover (Table 1). Vaccination rates among those with chronic lung disease were 10% higher among medical card holders (82%, 95% CI: 76-86%) compared to non-medical card holders (71%, 95% CI: 60-79%). Of those with congestive heart failure, 89% of those with a medical card were vaccinated (95% CI: 78-95%) compared to 69% of those without a medical card (95% CI: 46-85%).

**Table 1:** **Vaccination rates within each at-risk target group according to socio-economic enablers**

**Risk group** ^**a**^	**Vaccinated**							
	**Medical Card only**		**Dual cover**		**Private Health Insurance only**		**No additional cover**	
	**n**	**% (95% CI)** ^**b**^	**n**	**% (95% CI)** ^**b**^	**n**	**% (95% CI)** ^**b**^	**n**	**% (95% CI)** ^**b**^
Age ≥65 years^c^	1165	78.7 (76–81)	889	79.5 (77–82)	448	59.0 (55–63)	61	45.6 (37–55)
Chronic Heart Disease								
Angina	195	80.3 (75–85)	98	85.7 (77–91)	43	54.0 (42–65)	6	37.0 (16–64)
Heart Attack	155	81.4 (75–86)	80	83.8 (74–90)	43	54.3 (43–65)	9	47.8 (27–69)
Congestive heart failure	35	90.3 (76–96)	23	86.6 (66–96)	12	71.4 (45–88)	3	61.8 (19–92)
Abnormal heart rhythm	169	76.5 (70–82)	116	77.5 (70–84)	102	54.3 (46–62)	11	32.1 (19–49)
Other heart trouble	80	78.8 (69–86)	62	79.9 (68–88)	61	59.2 (48–69)	8	40.8 (23–61)
Chronic lung disease								
Chronic lung disease	131	79.1 (72–85)	60	88.1 (78–94)	51	71.5 (59–81)	18	68.2 (47–84)
Asthma	204	70.8 (65–76)	117	92.5 (86–96)	147	51.3 (45–58)	32	51.1 (38–64)
Diabetes	242	83.4 (78–87)	114	87.1 (80–92)	105	60.4 (53–68)	27	63.2 (47–77)
Chronic neurological disease								
Parkinson’s disease	16	94.6 (68–99)	11	100	6	44.7 (16–77)	1	42.3 (49–91)
Stroke	52	73.0 (60–83)	28	67.9 (51–81)	9	42.5 (22–66)	1	39.0 (5–89)
TIA	68	81.7 (71–89)	37	91.2 (79–97)	24	55.6 (39–71)	2	25.6 (5–60)
Chronic liver disease	13	62.4 (40–81)	7	67.5 (35–89)	7	54.0 (25–80)	0	0
BMI ≥40 kg/m^2^	44	67.0 (54–78)	16	80.7 (56–93)	18	38.2 (25–53)	8	51.5 (28–74)
Carer (in receipt of state allowance)	36	46.6 (35–58)	11	58.9 (35–79)	4	26.0 (10–54)	3	20.3 (6–52)
No risk factor	124	29.1 (25–34)	68	40.3 (32–49)	402	24.2 (22–27)	65	17.4 (14–22)
Burden of Chronic Illness								
None	157	41.3 (36–47)	92	50.5 (42–59)	174	20.4 (18–23)	36	13.3 (9–18)
High risk condition	835	75.6 (73–78)	481	83.1 (79–86)	425	52.2 (48–56)	86	45.3 (38–53)
Other Chronic illness	464	61.6 (58–65)	333	67.5 (63–72)	393	37.3 (34–41)	51	23.9 (18–31)
Cardiovascular risk factor	186	52.5 (47–58)	121	66.3 (58–74)	211	29.2 (26–33)	43	25.3 (19–33)
Fair/poor self-rated health	711	73.0 (70–76)	276	77.4 (73–82)	195	48.1 (43–53)	66	40.3 (33–48)
Total	1645	63.2 (61–65)	1030	71.6 (69–74)	1205	34.7 (33–37)	217	25.5 (22–29)

^*a*^ *Groups are not mutually exclusive as an individual may have more than 1 underlying health conditions listed.* ^*b*^ *Percentage estimates and confidence intervals adjusted for sample design.* ^*c*^ *At the time of data collection (2009), vaccination was typically recommended for those aged 65 and older. The national guidelines were updated in 2011 to include those aged 50 years and older.*

Table 2 presents the unadjusted and adjusted odds ratios describing the relationship between socio-economic enabling factors (medical card and private health insurance status) and vaccination rates. Vaccination was significantly associated with the level of entitlement to health services: 63.2% of those with a medical card reported being vaccinated (95% CI: 61-65%) compared to 25.5% of those with no additional cover (i.e. no medical card or private health insurance) (95% CI: 22-29%) (Table 2). In multivariate analysis, having a medical card was a significant independent predictor of vaccination, controlling for age, sex, income, self-rated health and clinical need (adjusted OR = 1.9, 95% CI: 1.5-2.5, p < 0.001). Having private health insurance only was not a significant predictor of vaccination (OR = 1.2, 95% CI: 0.9-1.5, p = 0.194). Stratified analysis by age, income and clinical need did not change the nature of the relationship between entitlement to healthcare and receipt of flu vaccination; having a medical card or dual coverage remained a significant independent predictor of vaccination (results not reported). Adjusted Population Attributable Fraction (PAF) estimates indicated that 8% of vaccination coverage was attributable to having a medical card (95% CI: 5-11%) (Table 2).

**Table 2:** **Association between socio-economic enablers and vaccination rates adjusted for demographic and need factors**

**Entitlement to health services**	**Vaccinated**						
	**N**	**% (95% CI)** ^**a**^	**Unadj. 0R (95% CI)**	**p value**	**Adj. OR** ^**b**^ **(95% CI)**	**p value**	**PAF(95%CI)** ^**c**^
No additional cover	217	25.5% (22–29)	1		1		
Medical card only	1645	63.2% (61–65)	5.0 (4.1-6.1)	0.000	2.0 (1.6-2.5)	0.000	0.08 (.05-.11)
Dual coverage	1030	71.6% (69–74)	7.4 (6.0-9.1)	0.000	2.2 (1.7-2.8)	0.000	0.05 (.03-.07)
Private Health Insurance only	1205	34.7% (33–37)	1.6 (1.3-1.9)	0.000	1.2 (1.0-1.5)	0.096	0.03 (−.01-.07)

^a^Percentage estimates and confidence intervals adjusted for sample design.

^b^Model adjusted for age (continuous) and sex, clinical need (including BMI > 40, carer status, guideline targeted chronic illness, other chronic illnesses, cardiovascular risk), self-rated health and income tertiles.

^c^Population attributable fraction for vaccination rates based on OR adjusted for all other variables.

## Discussion

In this study, older people with a medical card were twice as likely to be vaccinated against influenza, controlling for age and clinical need. Uptake rates were consistently higher among those with a medical card across all risk groups. In our study, 74% of those aged 65 years and older had received the flu vaccination in the past. However, given that the study assessed having *ever* received the flu vaccination, coverage in the most recent campaign is likely to be much lower.

The results suggest that the partial public reimbursement policy is leading to inequalities in vaccination coverage among those with established clinical need recommended to receive the flu vaccination. The findings suggest a step-like access to services in the Irish primary care system; those on the lowest incomes qualify for free access to services in general practice while those whose incomes are just above the threshold for medical card eligibility face difficulties accessing services, regardless of clinical need. The results may also reflect the wider influence of medical card status on behaviour. Medical card patients have significantly higher probability of visiting their GP compared to those with private insurance, even when differences in age and health status are considered [22]. A recent study estimated that people with a medical card aged 70 years and over attended almost 10 times a year on average [23]. In this study, private health insurance was not significantly associated with vaccination coverage illustrating the lack of additional benefit conferred by private health insurance in terms of accessing primary care services in Ireland.

Countries that reimburse healthcare practitioners for administering the vaccine or those that provide vaccination within their public health insurance schemes tend to have higher vaccination rates [6,24]. In Ireland, GPs are paid a nominal fee by the Health Service Executive for administering the vaccine to patients with a medical card. Results from the European SHARE project indicated that preventive service use was higher in health systems where doctors were paid a fee-for-service, however the effect was not significant for flu vaccination [7].

Advice from health care professionals has a significant impact on vaccination uptake [6,13,25,26], however it was not possible to control for this and other physician or system-related confounders in the analysis. Few studies have examined these factors which may contribute to variability in flu vaccination rates. A recent study by Blank *et al.* suggested an association between features of European vaccination policies and coverage rates in the elderly [27]. Countries which issued personal letters offering free vaccination showed higher coverage rates. This approach is not universally implemented in Ireland [27] and the practice may be restricted to those with a medical card who are easily identifiable within a practice computer system. Other organisational interventions shown to have a positive impact on vaccination rates include standing orders in the hospital or primary-care settings, a patient mailing system [28], patient reminder and recall systems in primary care settings [29] and providing the vaccination free of charge [30].

The association between age, chronic illness and vaccination coverage found in this study is well established in previous research [6,7,13-15]. Within the clinical risk group, we found that those in younger age group (50–64) had lower vaccination coverage (53%) than those in the older age group (81%) (≥65 years). While it could be argued that the older group have a longer lead-in time to ever have been vaccinated, there is evidence that age-based immunisation policies are associated with higher uptake among clinical risk groups than standard risk-based policies. In a study of vaccination uptake among clinical risk groups, Sammon *et al.* found that uptake was higher among age groups where universal flu vaccination was recommended compared to age groups in which only clinical groups were recommended for vaccination [5]. The Centers for Disease Control and Prevention (CDC) suggest that this may be due to the simpler health message attached to age-based policies, eliminating the need for GPs to identify high risk individuals [31]. It is estimated that implementing a 100% vaccination rate programme for all risk groups in the five biggest European countries would require an additional investment of €1.52 billion euro and would result in estimated savings of €39.45 million from reduced primary care visits and further savings of €1.59 billion from reduced hospitalisations [32]. The Irish government has proposed the introduction of universal health insurance including free primary care services. Plans to phase in this reform by providing free GP care to those with chronic illnesses in the first instance were replaced with proposals to start with children aged 6 years or younger. Our study highlights some of the potential pitfalls of a piecemeal approach to access based on income.

A nationally representative random sample participated in TILDA, based on stratified cluster sampling to minimize selection bias [19]. The main limitation of this study is the absence of information on the timing of vaccination, making it necessary to use having *ever* been vaccinated as the outcome measure. Studies in other countries examined coverage in the previous 12 months [7,33] or the last vaccination campaign [14]. It was not possible in this study to ascertain whether an individual was entitled to a medical card or whether he/she was in a high risk group at the time of vaccination. However, past insurance status has been found to be a significant predictor of current insurance status in Ireland [34]. Similarly, several studies have demonstrated the positive impact possession of a medical card has on GP use in Ireland [22,35]. While we cannot assert definitively that possession of a medical card now will increase the likelihood of vaccination now, the observed persistence in insurance status and numerous studies detailing the relationship between current medical card status and current GP use strongly indicate that this is a reasonable inference to draw from these results. This paper has important policy implications as it highlights the weaknesses of a health system where access to services is based on income rather than need. The pattern detected in this study endures after adjustment for age, income and clinical need.

Self-reported vaccination was above 70% for those with a medical card in high risk clinical groups, although this rate varied between illness groups. While a self-reported outcome measure is open to recall bias, studies have found high sensitivity and a high level of agreement between medical records and self-reported vaccination [36,37]. It was not possible to account for all high risk groups in the dataset, for example data were not recorded on renal disease and identification of carers was based on receipt of state allowance, which represents only a minority of carers in Ireland [38].

## Conclusions

In our study, having a medical card significantly increased the likelihood of having received the flu vaccine, independent of clinical need. This suggests that having free access is a stronger driver of uptake than clinical need. The mismatch between vaccination guidelines and reimbursement policy is creating gaps in coverage and unequal access to recommended services for high risk groups.
